# Holocene vegetation, fire and land use dynamics at Lake Svityaz, an agriculturally marginal site in northwestern Ukraine

**DOI:** 10.1007/s00334-021-00844-z

**Published:** 2021-06-21

**Authors:** Christoph Schwörer, Erika Gobet, Jacqueline F. N. van Leeuwen, Sarah Bögli, Rachel Imboden, W. O. van der Knaap, Nadezhda Kotova, Sergej Makhortykh, Willy Tinner

**Affiliations:** 1 Institute of Plant Sciences, University of Bern, Altenbergrain 21, 3013 Bern, Switzerland; 2 Oeschger Centre for Climate Change Research, University of Bern, Hochschulstraße 4, 3012 Bern, Switzerland; 3 Institute of Archaeology, National Academy of Sciences of Ukraine, 12 Geroiv Stalingrada prospekt, Kyiv, 04210 Ukraine

**Keywords:** Biodiversity, Climate change, Human impact, Macrofossils, Palaeoecology, Pollen analysis

## Abstract

Observing natural vegetation dynamics over the entire Holocene is difficult in Central Europe, due to pervasive and increasing human disturbance since the Neolithic. One strategy to minimize this limitation is to select a study site in an area that is marginal for agricultural activity. Here, we present a new sediment record from Lake Svityaz in northwestern Ukraine. We have reconstructed regional and local vegetation and fire dynamics since the Late Glacial using pollen, spores, macrofossils and charcoal. Boreal forest composed of *Pinus sylvestris* and *Betula* with continental *Larix decidua* and *Pinus cembra* established in the region around 13,450 cal bp, replacing an open, steppic landscape. The first temperate tree to expand was *Ulmus* at 11,800 cal bp, followed by *Quercus*, *Fraxinus excelsior, Tilia* and *Corylus* ca. 1,000 years later. Fire activity was highest during the Early Holocene, when summer solar insolation reached its maximum. *Carpinus betulus* and *Fagus sylvatica* established at ca. 6,000 cal bp, coinciding with the first indicators of agricultural activity in the region and a transient climatic shift to cooler and moister conditions. Human impact on the vegetation remained initially very low, only increasing during the Bronze Age, at ca. 3,400 cal bp. Large-scale forest openings and the establishment of the present-day cultural landscape occurred only during the past 500 years. The persistence of highly diverse mixed forest under absent or low anthropogenic disturbance until the Early Middle Ages corroborates the role of human impact in the impoverishment of temperate forests elsewhere in Central Europe. The preservation or reestablishment of such diverse forests may mitigate future climate change impacts, specifically by lowering fire risk under warmer and drier conditions.

**Supplementary Information:**

The online version contains supplementary material available at 10.1007/s00334-021-00844-z.

## Introduction

The Neolithic revolution, which established a sedentary lifestyle with agricultural products as the main food source in early human societies, had a significant impact on natural ecosystems in Central Europe. Not only did people in the Neolithic introduce new species such as crops or adventives (Behre 1981; Lang 1994), they also directly affected the species composition and structure of the existing natural forest by using fire to clear areas for arable farming, selective logging of tree species and browsing by domesticated animals (Lang 1994; Ralska-Jasiewiczowa et al. 2003; Tinner and Lotter 2006; Schwörer et al. 2015; Roberts et al. 2018; Rey et al. 2019). Such anthropogenic disturbance led to the widespread decline of disturbance-sensitive taxa such as *Tilia*, *Ulmus* or *Abies alba* in Central European forests and promoted disturbance-adapted trees, shrubs and herbaceous apophytes (Behre 1981; Birks and Tinner 2016; Rey et al. 2019). In the case of *Ulmus,* the rapid decline all over northwestern Europe in the mid-Holocene has also been attributed to the spread of a pathogen (Peglar 1993; Parker et al. 2002), although the remarkable coincidence with the Neolithic transition over large spatial scales indicates that humans might have played a role after all, possibly by indirectly facilitating the spread of the pathogen (Parker et al. 2002; Ralska-Jasiewiczowa et al. 2003). The increasing pressure of anthropogenic disturbance over millennia led to a decrease in tree species diversity, in some cases resulting in almost monospecific forests of disturbance-tolerant or actively favoured tree species such as *Picea abies* (Norway spruce) or *Larix decidua* (European larch) in the Alps (Gobet et al. 2003; Schwörer et al. 2015) or *Fagus sylvatica* (European beech)*, Quercus robur* (pedunculate oak) or *Pinus sylvestris* (Scots pine) in Central Europe (Ralska-Jasiewiczowa et al. 2003; Bobek et al. 2018; Rey et al. 2019).

From its origin in the Levant, the Neolithisation spread to Anatolia, then arrived around 8,800–8,500 calibrated years before present (cal bp) on the European mainland in Greece (Reingruber and Thissen 2009; Kotsakis 2014), and then advanced via the Balkans around 7,500 cal bp to Central Europe (Ammerman and Cavalli-Sforza 1971; Pinhasi et al. 2005; Tinner et al. 2007; Skoglund et al. 2012; Hofmanová et al. 2016; Gassner et al. 2020). However, other pathways have been proposed that better explain local archaeological findings, such as via islands in the Mediterranean Sea towards Northern Italy, Southern France and the Iberian Peninsula (Guilaine 2018), via the western Black Sea and the Dniestr river to northeastern Europe (Betti et al. 2020), or even via the Caucasus or eastern Black Sea coast to the Ukrainian steppes and onwards to Central Europe (Kotova 2009). Moreover, local domestication or cultivation efforts may have started already during the Mesolithic, before the full Neolithisation material package was introduced (e.g. Tinner et al. 2007; Miras et al. 2010; Lambert et al. 2019).

Since the spread of the Neolithic in Europe also coincided with major climatic changes after 8,200 cal bp (Tinner and Lotter 2006) and was in turn also influenced by climatic factors (Betti et al. 2020), it is difficult to determine if temperate forest dynamics are driven mostly by climate or anthropogenic impact, or a combination of both. Approaches that overcome these limitations include rigorous multi-proxy studies with independent lines of evidence, high-resolution time-series analyses (Tinner et al. 1999; Schwörer et al. 2015; Rey et al. 2019) or the combination of palaeoecological analyses with dynamic vegetation modelling (Heiri et al. 2006; Henne et al. 2013; Schwörer et al. 2014). Another approach is to study natural forest dynamics under low or absent human impact, for example in peripheral areas that were either too remote for agriculture or less well suited, e.g. due to poor or waterlogged soils. This applies to the western Polesie region in Central Europe, one of the largest forested regions of the continent with a low population density due to extensive marshes and nutrient-poor, sandy soils that prevent large-scale agriculture. By contrasting vegetation dynamics from marginal areas with sites with excellent conditions (e.g. fertile loess or morainic soils) that have been heavily impacted by human activity for millennia, we can assess the role of human disturbance and identify the near-natural vegetation under climatic conditions comparable to those of today and the recent past. Here, we present a novel vegetation reconstruction based on pollen, macrofossil and charcoal analyses from Lake Svityaz, at the border triangle of present-day Ukraine, Belarus and Poland. We are particularly interested in addressing the following research questions: (1) How did the vegetation react to climatic changes since the Late Glacial period; (2) What is the timing and impact of the first agricultural activities in our study area; (3) Can we use the Lake Svityaz sediment record to check if the impoverishment of forests in Central Europe is driven by human impact? Our results may help ecosystem managers to assess the potential natural vegetation of this area and maintain ecosystem services in a warmer and drier future.

## Methods

### Study site

**Fig. 1:**
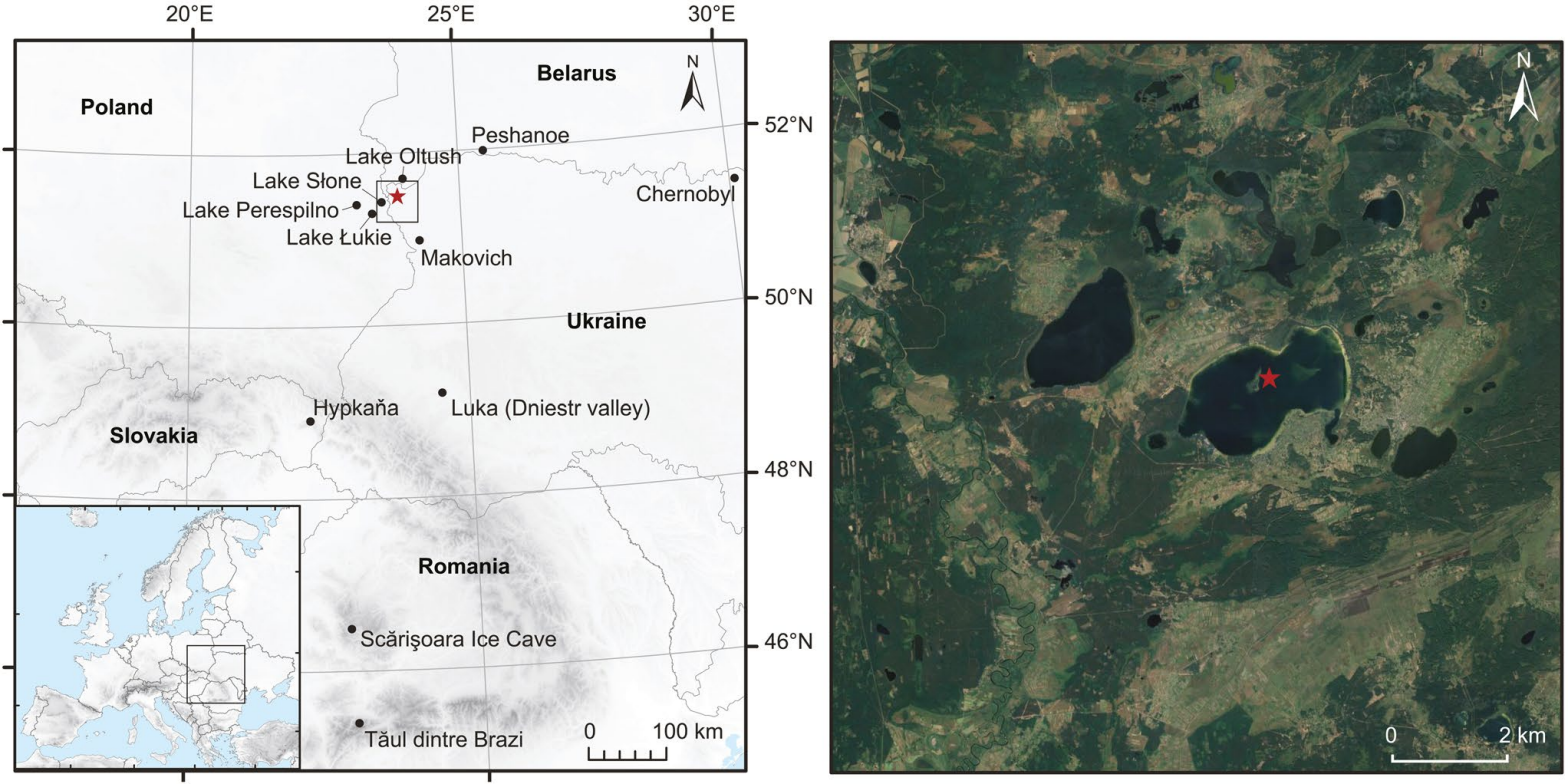
Location of the study site and other palaeoecological and palaeoclimatic records in Central Eastern Europe (left) and the coring location in Lake Svityaz (red star)

Lake Svityaz (Oзepo Cвiтязь) is the deepest lake in the Ukraine with a maximum water depth of 58.4 m. With a surface are of 25.2 km^2^ and a catchment area of 43.6 km^2^, it is also the second largest lake in the country and part of the Shatsk lake district, which is protected as a national park, a Ramsar site and a UNESCO biosphere reserve. The lake lies in the southwestern part of the Polesie region, within the Western Bug basin in the Volyn province, close to the borders of Poland and Belarus (Fig. 1). It is of karstic origin and is located ca. 300 km south of the range of the last glaciation. In 1887, the lake was connected to nearby Lake Luka by a 3 km long channel, which led to a lowering of the water table by 3 m (Tarasov et al. 1996). The geology of the area is characterized by Upper Cretaceous carbonate rocks, overlain by Quaternary glaciogenic and fluvial deposits such as sands and till (Dobrowolski et al. 2010, 2015). The topography is very flat with only minor elevation changes. The climate in the study area is temperate continental with warm summers, cool winters and rather low precipitation. Average July and January temperatures at the nearby weather station of Włodawa are 17.2 °C and  −4.1 °C respectively for the normal period 1981–2010. The mean annual precipitation is 500 mm with highest values during the summer months (Meteostat 2021). The vegetation in the study area is a mosaic of forests, pine plantations, marshes, peatlands and meadows. The forests are mixed temperate oak forests dominated by *Quercus robur* and *Pinus sylvestris* but also contain many other deciduous species such as *Betula pendula* (silver birch), *Fraxinus excelsior* (common ash), *Tilia cordata* (small-leaved linden) and *Carpinus betulus* (common hornbeam).

### Lake sediment coring and subsample preparation

In July 2011, we retrieved two parallel sediment cores with a UWITEC piston corer at a water depth of 12 m (Fig. 1; 51.505°N, 23.843°E; 157 m a.s.l.). We split the cores horizontally in the laboratory and assembled them to a composite master core of 12.3 m using visual correlation of distinct sediment layers. For pollen and microscopic charcoal analysis, we took 103 subsamples of 1 cm^3^ at regular intervals and processed these using standard procedures with HCl, KOH, HF, acetolysis and mounting in glycerine (Moore et al. 1991). To estimate pollen and charcoal concentration and influx we added *Lycopodium* tablets with a known number of spores prior to the chemical treatment (Stockmarr 1971). We identified pollen, spores and other non-pollen palynomorphs (NPP) under a light microscope at 400 × magnification using published keys (e.g. Moore et al. 1991; Reille 1999; Beug 2004) and the reference collection at the University of Bern. We counted a minimum number of 500 pollen grains excluding aquatic plants and spores (average 843 pollen grains) with the exception of two samples at the bottom of the core, where the pollen sum was 318 and 410. In total we identified 216 different pollen, spore and NPP-types. We also identified and counted plant stomata in the pollen slides using the key of Trautmann (1953) and the illustrations in Finsinger and Tinner (2020). Microscopic charcoal particles > 10 µm were counted on the pollen slides following Tinner and Hu (2003) and Finsinger and Tinner (2005). We subdivided the pollen data into statistically significant local pollen assemblage zones (LPAZ) using optimal partitioning with minimal sum-of-squares (Birks and Gordon 1985) and the broken-stick model (Bennett 1996) using the software R 3.5 (R Core Team 2015). For the macrofossil analysis, we took 75 subsamples with a thickness of 2 cm and a volume of 8–15 cm^3^ and sieved them with a mesh size of 200 µm. We identified plant remains under a stereomicroscope at 10–50 × magnification using published keys (e.g. Schoch et al. 1988), as well as the reference collection at the University of Bern. Macrofossil concentrations were standardized to a volume of 8 cm^3^.

### Chronology

Radiocarbon dating was carried out at the Poznan University Radiocarbon Laboratory and the Laboratory for the Analysis of Radiocarbon with AMS (LARA) at the University of Bern. The dates were calibrated to years before present (cal bp) using the IntCal13 calibration curve (Reimer et al. 2013). The age-depth model of Lake Svityaz was calculated based on 9 AMS radiocarbon dates from terrestrial plant remains (Table 1), using the program Clam (Blaauw 2010) with Monte Carlo sampling with 10,000 iterations and a monotonic spline function. Additionally, we calculated an extended 95% confidence envelope using mixed-effect modelling, taking into account the within object variance (calibration error) as well as the between object variance (sample thickness), according to Heegard et al. (2005).

**Table 1:** Radiocarbon dates and calibrated ages used to calculate the age–depth model of the Lake Svityaz sediment record

Depth (cm)	Lab. code	Material dated	^14^C-Age (year bp)	Cal. age, 2σ (cal bp)	Age in diagram (cal bp)
148–150	Poz-51308	Terrestrial leaf fragments, *Betula* S, coniferous BS	890 ± 60	922–703	815
281–285	Poz-51309	Terrestrial leaf fragments, *Betula* S, coniferous BS, deciduous P	2,610 ± 35	2,788–2,622	2,742
430–436	BE-6930.1.1	Terrestrial leaf fragments, *Betula* S & FS	4,345 ± 100	5,295–4,650	4,978
564–568	Poz-51310	Terrestrial leaf fragments, *Betula* S, coniferous P	5,520 ± 70	6,446–6,190	6,322
656–660	BE-6728.1.1	Terrestrial leaf fragments, *Betula* S, deciduous P	6,245 ± 65	7,301–6,983	7,149
750–754	BE-6727.1.1	Terrestrial leaf fragments, Betula S & FS	7,530 ± 65	8,424–8,194	8,328
867–869	Poz-51311	*Betula* S & FS, indet. S, coniferous BS, deciduous P	11,640 ± 50	13,575–13,371*	–
954–956	BE-6726.1.1	Twig indet., deciduous BS, *Betula* S, bud indet	10,455 ± 60	12,556–12,112	12,350
1,016–1,018	Poz-51307	Terrestrial leaf fragments, coniferous BS, deciduous P	11,290 ± 50	13,254–13,065	13,150

*S* seed, *BS* budscale, *P* periderm, *FS* fruitscale

^*^Rejected

### ﻿Numeri﻿cal analyses

To identify gradients in the vegetation composition over time, we used ordination analysis (ter Braak and Prentice 1988; Legendre and Birks 2012). We first analyzed the untransformed pollen data with a detrended correspondence analysis (DCA) using the software CANOCO 5.1 (ter Braak and Šmilauer 2017) to choose the appropriate response model (unimodal or linear). Since the gradient was rather short (1.9 SD) we decided to use a linear method, i.e. principal component analysis (PCA) with square-root transformation of the pollen percentage data.

To estimate biodiversity changes during the Holocene, we used palynological richness (PRI), the probability of interspecific encounter (PIE) and evenness-detrended palynological richness (DE-PRI). Palynological richness is the number of different pollen types per sample, calculated using rarefaction analysis with a constant pollen sum of 457 pollen grains per sample (Birks and Line 1992). PRI has been used by several studies to estimate past plant diversity in the landscape (Odgaard 1999; Colombaroli et al. 2013; Schwörer et al. 2015), but can be affected by differences in pollen productivity that modify the palynological evenness of a sample, compared to the evenness of the vegetation (van der Knaap 2009). To assess such potential biases, we calculated PIE according to Hurlbert (1971) and DE-PRI according to Colombaroli and Tinner (2013). We used the software R 3.5. (R Core Team 2015) with the package vegan (Oksanen et al. 2015) for all numerical analyses unless specified otherwise.

## Results and interpretation

### Lithology and chronology

**Fig. 2:**
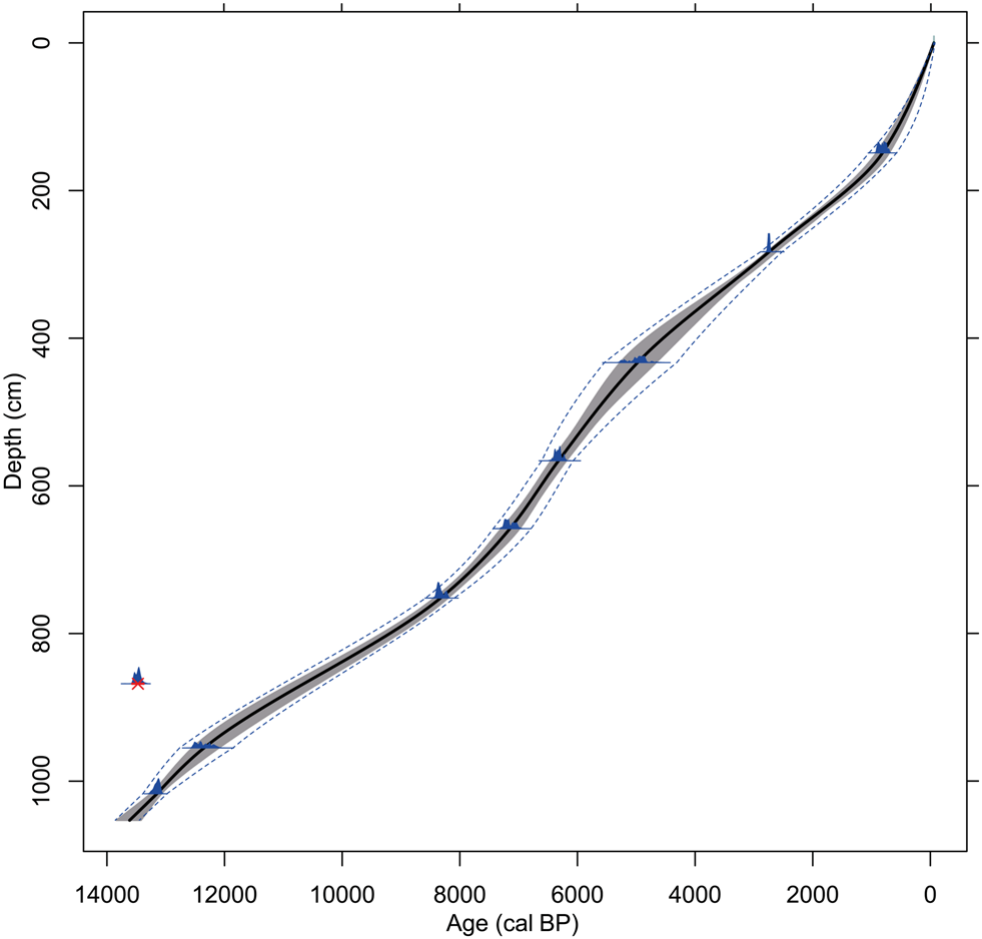
Age-depth model of the Lake Svityaz sediment record, based on eight calibrated radiocarbon dates (blue density curves). One radiocarbon date was rejected due to an unrealistic age (red cross). The grey area shows the 95% confidence interval of the age–depth model using Monte Carlo sampling with 10,000 iterations and a monotonic spline function. The dashed blue lines show 95% confidence intervals of a mixed-effect model, taking into account between-object variance (sample thickness; Heegard et al. 2005). The black line shows the best fit of the age–depth model that has been used for drawing the pollen diagram. The age–depth model was calculated using the program clam 2.2 (Blaauw 2010) with the IntCal13 calibration curve (Reimer et al. 2013)

In total, we retrieved 12.32 m of lake sediment. The lowermost sediment consists of lake marl (1,232–1,218 cm) and sand (1,218–1,054 cm) and did not contain any identifiable pollen or plant remains. Above 1,054 cm the sediment changes to silt and dark to light brown calcareous gyttja (1,050–0 cm). The 9 radiocarbon dates from our record span the Late Glacial to Holocene, with the oldest date at 13,255–13,065 cal bp (Table 1; Fig. 2). We had to reject one radiocarbon date at 868 cm since it is clearly too old and did not fit into the age-depth model. The sediment accumulation rate is fairly linear, with only small changes throughout the record, such as a slight increase from ca. 8,000–5,000 cal bp and during the last 500 years (Fig. 2).

### Pollen, macrofossil and charcoal analysis

We identified 8 statistically significant local pollen assemblage zones (LPAZ; SVI-1–8) that we use to discuss the pollen and macrofossil analysis.

**Fig. 3:**
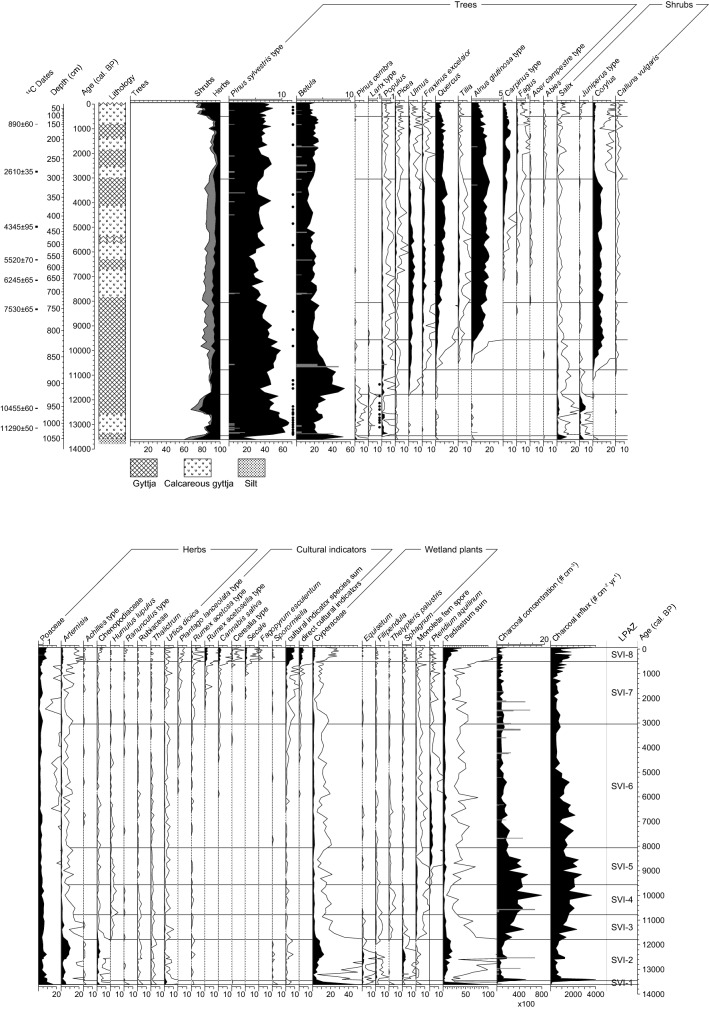
Combined pollen and macrofossil diagram of selected taxa, including lithology, spores, stomata and microscopic charcoal concentration and influx. Direct cultural indicators include only crops, i.e. *Cannabis sativa* and cereals. *LPAZ* local pollen assemblage zone. Empty curves show 10 × exaggeration. Grey bars indicate total macrofossil concentrations (for 8 cm^3^) on the scale given at the top of the diagram. Black dots show presence of stomata

SVI-1 (13,600–13,450 cal bp): The oldest LPAZ has the lowest arboreal pollen (AP) percentages of the entire record, ca. 60% (Fig. 3). Wind-pollinated *Betula* is the dominant pollen reaching up to 50%. Rather high values are also reached by *Juniperus*-type (5%) and *Salix* (10%)*.* Non arboreal pollen (NAP) percentages reach ca. 30% and are dominated by Poaceae and *Artemisia*, with considerable shares of Chenopodiaceae and *Urtica dioica*. Cyperaceae and remains of the green algae *Pediastrum* spp. reach the highest values of the record. No arboreal macrofossils were found in this LPAZ and only very few, unidentified plant remains (Figs. 3 and ESM). Microscopic charcoal concentration and influx are very low, whereas macroscopic charcoal has been found, although in low quantities. The low AP percentages as well as the absence of arboreal macrofossils suggest a very open landscape surrounding the lake, dominated by steppic elements such as *Artemisia* and Chenopodiaceae and likely scattered *Betula*, *Salix, Juniperus* and possibly *Pinus sylvestris* trees or shrubs. Burning was rather rare in the steppic environments, likely due to low standing biomass. The maximum of *Urtica dioica* may have been caused by nitrogen-rich environments, while high abundances of Cyperaceae and *Pediastrum* may have resulted from low water tables.

SVI-2 (13,450–11,800 cal bp): AP rises to almost 90% at the beginning of this zone, before dropping to ca. 80% in the second part. The zone is dominated by *Pinus sylvestris*-type, with up to 65% of the pollen sum, as well as *Betula* with ca. 20%. Also, other boreal and montane tree pollen is present, specifically *Pinus cembra*, *Larix*-type and *Picea*. Cold, steppic taxa like *Juniperus*-type, *Artemisia* and Chenopodiaceae show distinct pollen peaks in the second part of the zone, when AP declines (ca. 12,600–11,800 cal bp). Spores of the coprophilous fungi *Sporormiella* are present, albeit in low numbers. Arboreal macrofossils and stomata document the local presence of *Pinus sylvestris,* tree *Betula* (*B. pubescens* or *B. pendula*), tree *Alnus* (*A. glutinosa* or *A. incana*), *Larix decidua* and *B. nana*. Charcoal concentration and influx show a marked peak at the beginning of the zone but then decline to relatively low values, suggesting a gradually decreasing regional fire activity. The marked increase in AP, as well as the first trees documented with macrofossils around 13,450 cal bp, indicate an expansion of closed, boreal forest in the region. The marked decrease in AP and an increase of steppic elements between 12,600 and 11,800 cal bp points to a reversal to open forest steppe.

SVI-3 (11,800–10,800 cal bp): AP rises again to ca. 90% and stays at this level. Notable is the marked increase of *Betula* pollen to 40–55%, some of the highest values of the record. This zone has the first continuous presence (empirical limit) of temperate taxa such as *Ulmus*, *Fraxinus*, *Corylus* and *Quercus*, although the latter three in low values. We did not analyze any macrofossil samples in this zone due to the low sampling resolution. Microscopic charcoal concentration and influx gradually increase during SVI-3 with a marked peak at ca. 11,380 cal bp. The results indicate closed forest with temperate trees such as *Ulmus* expanding in the surroundings of Lake Svityaz. Concomitantly, regional fire activity gradually increased.

SVI-4 (10,800–9,550 cal bp): Tree pollen stays high at ca. 90%. *Betula* gradually decreases to ca. 25%, whereas several temperate taxa such as *Ulmus*, *Fraxinus*, *Quercus* and *Corylus* increase, the latter rapidly and massively (rational limit). *Tilia* and *A. glutinosa*-type reach their empirical limit and are continuously present. Microscopic charcoal concentration and influx still increase with another marked peak at ca. 10,000 cal bp. Temperate tree pollen suggests that forest composition changed to a mixed deciduous forest, although with abundant boreal elements such as *P. sylvestris* and *Betula.* Forest fire activity reached a temporary maximum that was regained only during the past 500 years.

SVI-5 (9,550–8,050 cal bp): AP stabilizes at very high values (> 90%), mostly consisting of *P. sylvestris*-type, *Betula, A. glutinosa*-type and *Corylus*. Pollen of temperate taxa such as *Ulmus*, *Quercus* and *Tilia* is still increasing, whereas *P. sylvestris*-type is gradually decreasing to ca. 40%. *A. glutinosa*-type shows a drastic increase to ca. 20%. *Pteridium aquilinum* reaches the empirical limit and markedly increases after ca. 8,800 cal bp. Microscopic charcoal concentration and influx are still very high at the beginning of this zone, before dropping at the end, suggesting that fire activity remained high, only to decrease at ca. 8,200 cal bp. In general, vegetation structure and composition remained comparable to that of the previous zone, with the difference that the mass expansion of *A. glutinosa*-type most likely points to the establishment of alder carr vegetation around the edge of the lake and in marshy areas in the region.

SVI-6 (8,050–3,050 cal bp): Throughout the zone, AP remains consistently very high (> 90%). All temperate tree taxa show an optimum with the highest values in this zone. *Carpinus*-type, *Acer* and *Fagus* show the first continuous presence at ca. 7,200 and 6,000 cal bp respectively, the latter after its first appearance around 8,400 cal bp. Cultural pollen indicators, such as *Plantago lanceolata*-type*, Cannabis sativa* and Cerealia-type occur for the first time after ca. 5,800 cal bp, although only intermittently or with very low values. Macrofossils and stomata document the local presence of *P. sylvestris*, *Betula* and *Alnus*. Microscopic charcoal concentrations and influx gradually decrease, suggesting declining fire activity. Closed temperate-continental forests dominated by *Quercus*, *Ulmus*, *Fraxinus excelsior*, *Tilia*, *Corylus avellana* and *P. sylvestris* surrounded Lake Svityaz until ca. 6,000 cal bp. Subsequently, *Carpinus betulus* and oceanic trees such as *Fagus sylvatica* and after 4,000 cal bp also *Abies alba* expanded. Very low and fragmentary occurrences of anthropogenic pollen indicators suggest weak farming activity in the area but with no discernible impact on the forest vegetation.

SVI-7 (3,050–500 cal bp): AP shows distinct fluctuations but stays generally above 90%. Several temperate tree species such as *Ulmus*, *Fraxinus*, *Tilia* and *Corylus* show strong decreases in pollen percentages at the beginning of this zone*.* On the other hand other deciduous taxa increase such as *Carpinus*-type and *Fagus* or stay at high values (e.g. *Quercus* and *A. glutinosa*-type). The local presence of *F. sylvatica* near Lake Svityaz is documented by a single budscale in the macrofossil record. Cultural pollen indicators such as *Cannabis sativa*, *Rumex acetosella*-type and *P. lanceolata*-type are continuously present but remain at low values. However, pollen of crops such as Cerealia-type and *Secale* occur only intermittently. Microscopic charcoal concentration and influx remain relatively low, but macroscopic charcoal concentrations show very high peaks. Pollen and charcoal analyses indicate some small scale, short-lived, anthropogenic forest openings and a reduction of disturbance-sensitive and/or light-demanding temperate trees (*Tilia*, *Fraxinus excelsior*, *Acer*, *Ulmus*) and shrubs (*Corylus avellana*) in the forests surrounding the lake, while the rather disturbance-resistant *Quercus*, *C. betulus*, *F. sylvatica* and *A. glutinosa* remained fairly stable or even expanded.

SVI-8 (500 cal bp–present): At the beginning of this zone, AP drastically decreases to ca. 80%, whereas herbs (NAP) increase. All tree taxa were equally affected, while light-demanding shrubs (e.g. *Salix, Juniperus* type, *Corylus*) expanded. Cultural pollen indicators such as *Rumex acetosella*-type*, Cannabis sativa*, Cerealia-type and *Secale*, as well as Poaceae show marked increases. The alga *Pediastrum* increases to its highest values at the end of the record. Microscopic charcoal influx increases, whereas its concentration remains low. The decrease in AP and the increase in anthropogenic pollen indicators points to large-scale forest openings for agriculture, leading to a higher nutrient input into the lake. The last 500 years of the record documents the establishment of the present-day cultural landscape, characterized by a mosaic of forest, marshes and agricultural fields.

### Ordination and biodiversity

**Fig. 4:**
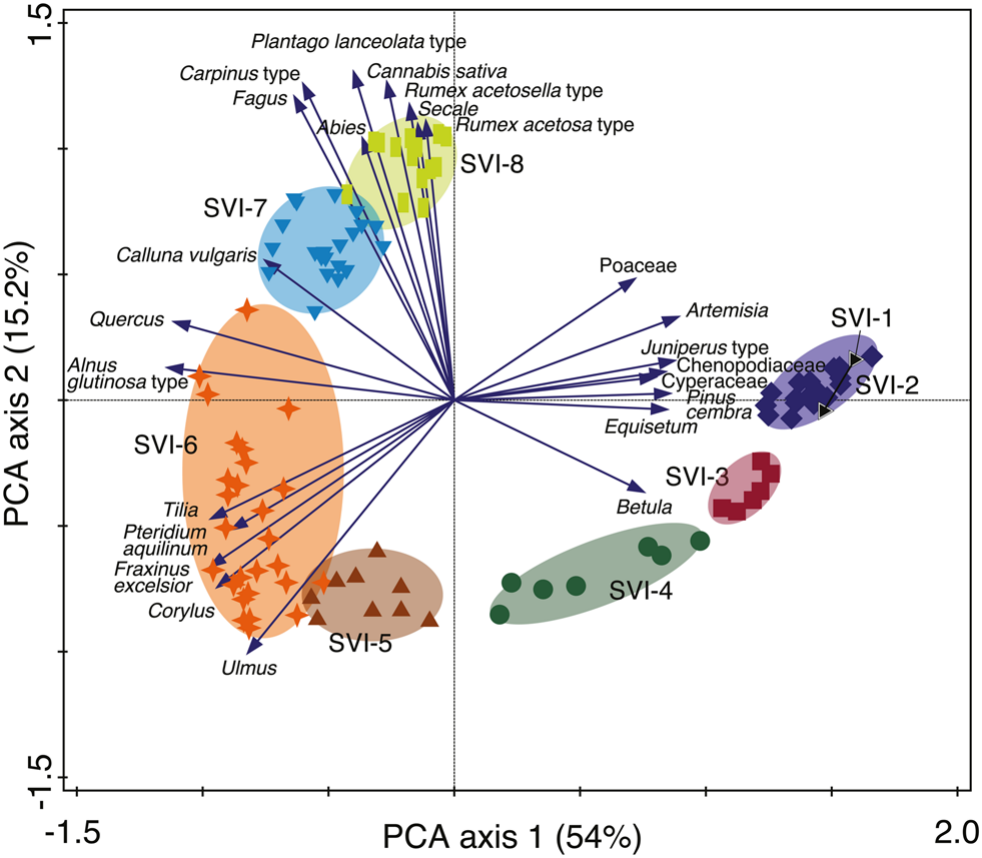
PCA biplot showing species and sample scores of the Lake Svityaz pollen record. PCA axis 1 represents a climatic gradient from temperate arboreal taxa with low scores to boreal and steppic taxa with high scores. PCA axis 2 indicates a gradient of anthropogenic disturbance, with human pollen indicators and taxa expanding in the Late Holocene having high scores and taxa from closed natural forest with low scores. The sample groups are based on the statistically significant pollen zones, showing a transition from open, steppic landscapes to closed forest and on to the present-day agricultural landscape

PCA axis 1 explains 54% of the variance in the pollen data and shows a climatic gradient, with temperate taxa such as *Quercus*, *Fraxinus excelsior, A. glutinosa*-type, *Corylus*, *Ulmus* and *Tilia* having low axis 1 scores, whereas boreal and steppic taxa such as *Pinus cembra, Juniperus*-type, *Betula, Artemisia*, Poaceae and Chenopodiaceae have high axis 1 scores (Fig. 4). PCA axis 2 explains 15% of the variance in the data and is related to anthropogenic activity with human pollen indicators such as *P. lanceolata*-type, *Cannabis sativa*, *R. acetosella*-type and *Secale* having high scores and trees such as *Ulmus, Corylus*, *Fraxinus*, *Tilia* and *Betula* having low scores. The low scores of the trees *Carpinus*-type, *Fagus* and *Abies* are explained by their late Holocene expansion (LPAZ SVI-7), when human impact also increased.

**Fig. 5:**
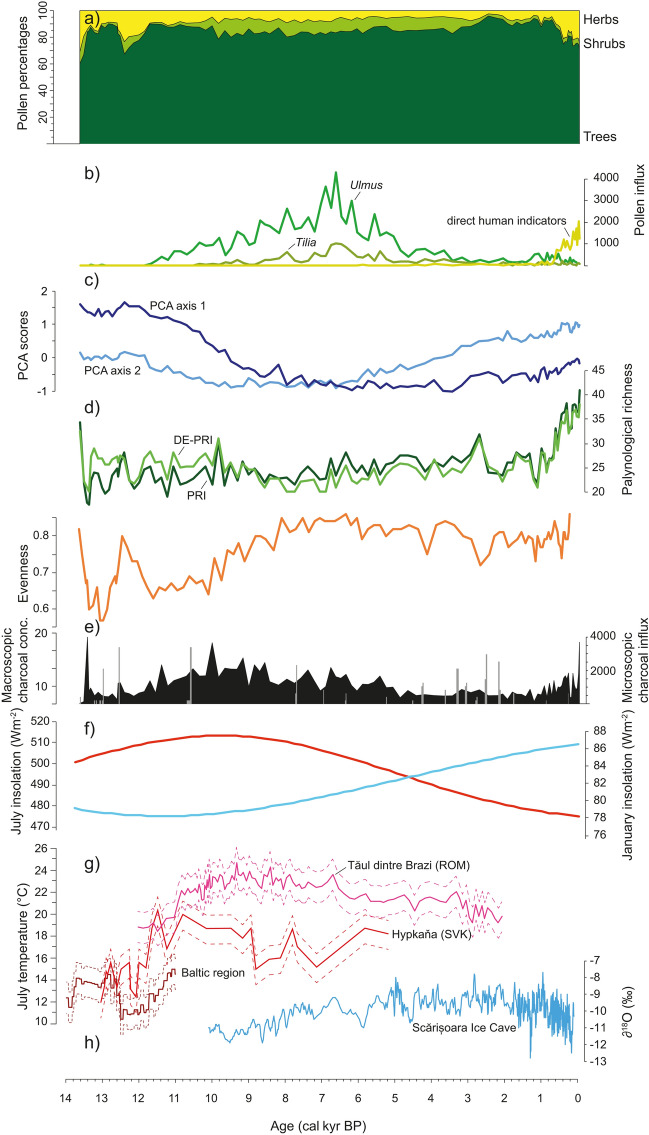
Comparison of biotic proxies from Lake Svityaz with insolation and regional climate reconstructions: **a** Summary diagram of the pollen analysis showing total tree, shrub and herb pollen percentages; **b** pollen influx (grains cm^−2^ year^−1^) of *Ulmus, Tilia* and the sum of direct human pollen indicators; **c** sample scores of PCA axes 1 and 2, respectively representing climatic and anthropogenic gradients of vegetation change; **d** palynological richness (PRI), evenness-detrended palynological richness (DE-PRI) and evenness of the Lake Svityaz pollen assemblage as biodiversity measures; **e** microscopic charcoal influx (black curve) and macroscopic charcoal concentrations (grey bars); **f** July (red) and January (blue) insolation at 51°N (Laskar et al. 2004); **g** chironomid-inferred July temperatures at sea level from the Baltic region (Heiri et al. 2014), Hypkana in eastern Slovakia (Hájková et al. 2016) and Tăul dintre Brazi, in the southern Carpathians of Romania (Tóth et al. 2015); and h) δ^18^O from the Scărișoara Ice Cave in the Apuseni Mountains as a proxy for autumn through early winter temperatures (Perșoiu et al. 2017)

Both palynological richness (PRI) and evenness-detrended palynological richness (DE-PRI) show no clear trend throughout the record, except for the last 1,000 years when both indices markedly increase, to reach the highest diversity in the most recent samples (Fig. 5). Palynological evenness (PIE) on the other hand shows large fluctuations at the beginning of the record, with a tendency to uneven conditions during periods of *Pinus* dominance (ca. 13,500–12,600 cal bp and 11,600–10,000 cal bp), before gradually increasing and stabilizing at high values after ca. 8,000 cal bp.

## Discussion

### Climatic drivers of vegetation dynamics

The lithology (inorganic silt) and pollen analysis (NAP > 20%) of the basal sediments of Lake Svityaz suggest that the record may extend to the Bølling or even Oldest Dryas prior to ca. 14,700 cal bp (van Raden et al. 2013). Unfortunately, we could not confirm this with radiocarbon dating, since we did not find any terrestrial macrofossils suitable for dating in this part of the sediment core. Nevertheless, such an old age would agree well with the onset of sediment accumulation in other lakes in the area. Both Lake Łukie and Perespilno in the Polish Polesie and Lake Słone in the Lublin Upland have been dated back to the Late Glacial (Bałaga 2004; Kulesza et al. 2011; Zawiska et al. 2015). The lithological change to organic gyttja at 1,050 cm in our core (tentatively dated to ca. 13,400 cal bp) coincides with a drastic increase in AP and the first occurrence of terrestrial macrofossils, indicating rapid forest expansion during the Bølling–Allerød interstadial (14,685–12,650 cal bp). At the beginning of the interstadial, temperatures increased by 3–6 °C within a century in Central Europe (Brooks and Heiri 2013; von Grafenstein et al. 2013; Heiri et al. 2014), leading to large-scale reorganization of terrestrial and aquatic ecosystems (e.g. Ammann et al. 2013; Feurdean et al. 2014; Zawiska et al. 2015). At Lake Svityaz, the pronounced warming at the beginning of the Bølling gradually led to the establishment of boreal forest dominated by *P. sylvestris* and *Betula* at ca. 13,450 cal bp. Interestingly, *P. cembra* and *Larix decidua* were also locally present during this period, as documented by the continuous presence of pollen as well as stomata and a single needle in the case of *L. decidua*. This suggests a close proximity to refugia where these species persisted during the coldest periods of the Last Glacial Maximum (LGM). Indeed, pollen and macrofossil findings indicate that the closest refugia were located in the forelands of the Carpathians (Lang 1994; Wagner et al. 2015), ca. 300 km to the south. However, given that our site was unglaciated, we cannot exclude that these very cold-tolerant trees survived the LGM locally in sheltered micro-habitats, where water availability was sufficient for tree growth. During the Late Glacial, *L. decidua* and *P. cembra* expanded northwards into the Central European lowlands (Lang 1994; Wagner et al. 2015). At present, the closest populations of *P. cembra* are situated in isolated high-elevation areas in the western Carpathians (Caudullo and de Rigo 2016). The present-day range of *L. decidua* also includes the Carpathians as well as the southeastern Polish lowlands (Da Ronch et al. 2016).

During the Younger Dryas cold period (12,650–11,700 cal bp; Engels et al. 2016) forests became more open around Lake Svityaz and steppic taxa increased, leading to the establishment of a forest-steppe. Vegetation-independent climate reconstructions in the region indicate an abrupt cooling of 1–4 °C in the summer months (Płóciennik et al. 2011; Heiri et al. 2014). The opening of the forests and the expansion of steppic elements can probably also be attributed to drier and more continental conditions (Pawłowski et al. 2015). With the beginning of the Holocene, forests closed again and the species composition gradually changed from a boreal forest dominated by *Betula* and *P. sylvestris* to mixed temperate deciduous forest dominated by *Quercus*, *Corylus*, *Tilia* and *Ulmus*. This drastic change in vegetation structure and composition can be attributed to a rapid warming of ca. 2–4 °C during the Younger Dryas-Holocene transition (Płóciennik et al. 2011; Feurdean et al. 2014; Heiri et al. 2014; Tóth et al. 2015; Hájková et al. 2016). The remarkable agreement between the chironomid-inferred July temperatures from the Baltic region (Heiri et al. 2014) and the tree pollen percentages at Lake Svityaz during the Younger Dryas (Fig. 5), indicate a strong impact of climate change on vegetation.

The first temperate tree to expand in the study area was *Ulmus* at ca. 11,800 cal bp, right at the end of the Younger Dryas. Its mass expansion was accomplished within a few centuries. This is remarkable since other temperate trees such as *Quercus*, *Tilia* or *Corylus,* expanded only ca. 500–1,000 years later in the area, although some were possibly locally present since the onset of the Holocene (e.g. empirical limit of *Quercus* at ca. 11,800 cal bp). Indeed, a recent study from Luka in the Dniestr Valley, Western Ukraine, found evidence for the local presence of *Ulmus* during the Late Younger Dryas (Kołaczek et al. 2018), whereas in the southern Carpathians, *Ulmus* was already locally present earlier (Feurdean et al. 2012), suggesting close proximity to a glacial refugium. From its refugium close to the southern Carpathians, *Ulmus* was apparently able to rapidly move northwards, possibly circumventing the Carpathian mountain range on the eastern side using river valleys such as the Dniestr as corridors (Kołaczek et al. 2018).

After ca. 10,800 cal bp the boreo-nemoral forest around Lake Svityaz with few temperate elements (*Ulmus* and likely also *Fraxinus, Quercus* and *Corylus* in low abundances) was progressively replaced by mixed-deciduous temperate forest characterized by *Quercus, Ulmus, Tilia, Corylus* and *Fraxinus*. Our ordination analysis identifies this shift in forest types as the most important vegetation change at Lake Svityaz in the last 14,000 years. PCA axis 1 scores over time show that the shift from boreo-nemoral to mixed temperate forest took place over a very long period of 3,000 years from ca. 11,000–8,000 cal bp (with an acceleration at 10,000 cal bp; Fig. 5). Climate reconstructions indicate that summer temperatures during the Early Holocene in eastern Central Europe were slightly warmer than today (Feurdean et al. 2014; Tóth et al. 2015; Hájková et al. 2016), mainly in response to a high summer insolation (Fig. 5). Pollen percentages but also influx values show the highest abundance of temperate trees such as *Ulmus*, *Tilia* and *Quercus* between 7,000 and 6,000 cal bp (Figs. 3 and 5).

### Climatic and biotic drivers of fire regimes

The steady increase of regional fire activity during the Early Holocene mirrors the summer insolation curve (Fig. 5), indicating that fire regimes were directly controlled by summer temperature and/or precipitation, which in turn also led to high forest productivity and therefore fuel availability. Indeed, the highest fire activity during ca. 10,500–8,500 cal bp coincides with the highest reconstructed summer temperatures during the Holocene in Central Europe (Tóth et al. 2015; Hájková et al. 2016). Interestingly, high fire activity did not prevent the establishment and population expansion of fire sensitive taxa such as *Tilia* and *Ulmus* in the region. This apparent discrepancy has also been noted in other studies in the larger surroundings, the Carpathians and the Dniestr valley (Hájková et al. 2016; Kołaczek et al. 2018, 2020), where it has been attributed to landscape heterogeneity, with fire-sensitive taxa occurring in wetter, less fire-prone habitats (Kołaczek et al. 2020). Therefore, fires around Lake Svityaz were likely restricted to drier areas on sandy soils dominated by *P. sylvestris* and did not affect vegetation in the wetter areas*.* In addition, charcoal influx indicates that fire activity was very modest compared to fire-prone southern Europe, likely not exceeding the tolerance of fire-sensitive species (maximum Holocene charcoal influx peak of 4,000 particles cm^−2^ year^−1^ vs. peaks of 40,000–400,000 particles cm^−2^ year^−1^ in southern Europe; Tinner et al. 2005, 2009, 2016). Regional fire activity slightly decreased after ca. 8,200 cal bp with decreasing summer insolation and a change to more oceanic conditions with milder winters (Perșoiu et al. 2017). Fire activity further declined after the regional expansion of broad-leaved deciduous forests dominated by *Carpinus betulus*, possibly even with admixed *Fagus sylvatica*, suggesting a biotic control of fire regimes. Recent studies from Central and Eastern Europe have argued that expansion of broad-leaved deciduous forest can lead to fire suppression due to lower flammability, higher forest density and moister microclimate (Feurdean et al. 2017; Bobek et al. 2019; Carter et al. 2020). However, it is difficult to disentangle climatic and biotic drivers of fire regimes at our site, since the expansion of mesophilous beech forests in Central Europe has been attributed to cool and wet conditions (Tinner and Lotter 2006; Giesecke et al. 2011) that in turn also have an effect on fuel flammability and fire spread. Consistently, the *F. sylvatica* expansion around 5,900 cal bp at Lake Svityaz falls in the cool and wet period from ca. 6,100 to 5,650 cal bp (Central European cold phase CE-5, see Haas et al. 1998; Heiri et al. 2004), when the tree expanded also elsewhere in eastern Central Europe (Lang 1994; Tinner and Lotter 2006).

### Human impact on pristine forests

Mesolithic people of the Komornicka and Janisławicka cultures were already present in the Polesie area since the Late Glacial, as evidenced by findings of flint tools (Bałaga 2007a). However, the impact of these hunter-gatherers on the natural vegetation was only marginal. First farming communities connected to sites of the Volynskaya Neolithic culture and Linear Pottery Culture (LBK) established in the second part of the sixth millennium bc in the region (Kotova 2003). Earliest archaeological findings of LBK from southeastern Poland and northwestern Ukraine have been dated to 5300–4800 bc or 7,300–6,800 cal bp, indicating a rapid eastward spread of the LBK (Kotova 2003; Motuzaité Matuzevičiūtė and Telizhenko 2016; Czekaj-Zastawny et al. 2020). In the northwestern Pontic region of the Ukrainian steppe, there is even earlier evidence for plant and animal domestication that has been attributed to the Bug-Dniestr culture, dated to the second half of the seventh millennium bc (ca. 8,200–8,000 cal bp; Telegin et al. 2003; Vinogradova and Kiosak 2017). There is no evidence of these earliest agricultural activities in the Lake Svityaz record, probably due to very low population densities and extremely localized impact on the vegetation. The first signs of agricultural activity by Neolithic societies in our record date to ca. 5,900–5,700 cal bp. This is evidenced by the first occurrence of pollen of *P. lanceolata *type and *Cannabis sativa*, two cultural indicators of agricultural fields (Behre 1981). This timing agrees well with other palaeoecological studies in the Belarusian Polesie region that date the beginning of the Neolithic based on palynological records to ca. 6,600–6,000 cal bp (Zernitskaya and Mikhailov 2009). During the time of the earliest palynological evidence of agricultural activity around Lake Svityaz (5,900–5,700 cal bp), people of the Lublin-Volynian Eneolithic culture settled in this region (Zakościelna and Starkova 2018). These people used more sophisticated agricultural practices than the LBK and focused on the cultivation of grain crops on the plateau. They also used fire as a tool of agricultural technology, not only to clear forests, but also to prevent scrub encroachment (Kadrow 2016). Nevertheless, our pollen record from Lake Svityaz indicates that population density was still very low, reaffirming the marginal nature of the area. However, the onset of agriculture in the region broadly coincides with both a population decline of disturbance-sensitive tree species such as *Tilia* and *Ulmus* around 6,500–5,800 cal bp and the regional establishment of *C. betulus* and *F. sylvatica* at 7,000 cal bp and 5,900 cal bp, respectively (empirical limits; Figs. 3 and 5). Both the decline of *Tilia* and *Ulmus* as well as the dominance of *F. sylvatica* and *C. betulus* in Central European forests have previously been attributed to human disturbance (Küster 1997; Ralska-Jasiewiczowa et al. 2003; Thöle et al. 2016; Rey et al. 2019). However, this does not exclude a prominent role of climatic change e.g. as resulting from decadal-scale oscillations or due to the millennial-scale effects of decreasing summer insolation (Fig. 5), given that the latter resulted in a shift to more oceanic conditions during the mid-Holocene. Most likely, the slight, temporary declines of *Tilia* and *Ulmus* at 6,500–5,800 cal bp were a result of competition with the more shade-tolerant *C. betulus* and later *F. sylvativa*, and not directly caused by human impact. At the end of the mid-Holocene, around ca. 5,000 cal bp, forests were composed of *C. betulus*, *F. sylvatica*, *Acer*, *P. sylvestris*, *Betula*, *Quercus*, *Ulmus*, *Tilia*, *Fraxinus excelsior* and *Corylus avellana*, thus displaying a very high diversity, which is also reflected in high evenness values (Fig. 5).

At Lake Svityaz, *F. sylvatica* reached the easternmost limit of its maximum range. Although currently the tree is not present anymore in the area (Houston Durrant et al. 2016), the occurrence of a single bud scale in the macrofossil record as well as the continuous curve in the pollen diagram points to its local presence around our study site. *Fagus* pollen also reaches the empirical limit in other sites in the area, such as at ca. 6,000 cal bp at Peshanoe and nearby Lake Oltush in southern Belarus (Zernitskaya 1991), during the mid-Holocene at several lakes in the Polish Polesie (Bałaga 2004, 2007b), or around 3,600 cal bp at Makovich in the Western Ukraine (Artushenko 1957). Unlike *C. betulus*, which is more tolerant of late frosts and an important component of the mixed temperate forests in the area, *F. sylvatica* was most likely only present in low numbers. Since the trees growing at Lake Svityaz represented the outermost edge of its range, they were most likely highly stressed, so that small changes in climate, such as the cooling during the Little Ice Age (Büntgen et al. 2011; Perșoiu et al. 2017) or increasing disturbance, might have been enough to lead to its local extinction.

With the intensification of land use in the Late Bronze Age (3,400 cal bp) linked to the Trzciniec Bronze culture (Czebreszuk et al. 1998), some forest trees such as *Ulmus*, *Tilia*, *Fraxinus* and *Corylus* abruptly declined in the surrounding area. High concentrations of macroscopic charcoal and the first presence of Cerealia-type pollen indicate that fire was used to create local, small scale forest openings for agriculture, likely close to the lake shore. The decline of *Corylus* is surprising, since it can resprout quickly, thrives after fire disturbance (Tinner et al. 1999) and is only affected by very frequent fires (Delarze et al. 1992). This sudden decline, which is accompanied by a decline in *Fraxinus* and is also evident in pollen influx, may be attributed to the further local expansion of the shade-tolerant *C. betulus* and *F. sylvatica* in the forest. A similar pattern has been observed at other lakes in the Polish Polesie and northern Poland, indicating regional-scale changes in the forest composition around 3,000 cal bp (Ralska-Jasiewiczowa et al. 2003; Bałaga 2007a, b; Lamentowicz et al. 2019). At the same time, a northward expansion of forest-steppe associated with climate aridification has been recorded in the European part of Russia (Spiridonova and Lavrushin 1997). Such synchronous vegetation changes over large spatial scales are commonly attributed to a climatic driver, since human disturbance would likely produce a more heterogeneous signal (Ralska-Jasiewiczowa et al. 2003; Jalut et al. 2009). However, climate can also trigger societal and demographic changes that lead to broad-scale synchronous patterns of human activity (Rey et al. 2019; Walsh et al. 2019). Even though human impact was still relatively low, the creation of open areas and increased disturbance led to a marked increase in palynological diversity (Fig. 5). Forests quickly recovered from local anthropogenic activities during the Late Bronze Age and overall regional forest cover was still very high.

During the Iron Age, around 2,400 cal bp, tree pollen even reached its highest values of the entire record (96%), whereas cultural pollen indicators and Poaceae markedly declined, indicating local land abandonment and closed, dense forests around the site, possibly in response to a short-lived cool and humid period (Haas et al. 1998; Büntgen et al. 2011). Later on, still during the Iron Age, there is again evidence for small-scale forest openings and agricultural activity around Lake Svityaz, as documented by a decline in tree abundance of *P. sylvestris* or *C. betulus*, and an increase in cultural pollen indicators, the occurrence of *Secale* and other cereal pollen (Fig. 3) and evidence for local fires. Increasing human impact at ca. 2,000 cal bp coincides with the southward expansion of Gothic tribes in the first and second century ad (Stolarek et al. 2019). During the subsequent migration period, land use pressure declined and forests quickly became closed again. Large-scale forest clearing and the establishment of the present-day cultural landscape only occurred during the last 500 years. This is reflected in a drastic increase of cultural pollen indicators such as Cerealia and *Secale* and open land taxa (e.g. Poaceae; Fig. 3), as well as in palynological richness (Fig. 5). Our data indicates that people preferentially cleared the broad-leaved deciduous forest and not drier and/or sandier areas dominated by *P. sylvestris*, to create fields mainly for arable farming with only little animal husbandry.

Overall, except for the last few 100 years of the record, human impact on the natural landscape was very low. Indeed, PCA axis 2, which reflects the gradient of natural forests to anthropogenic environments, only explains 15% of the variation in the dataset, in contrast to PCA axis 1 that explains 54% and likely reflects the climate-driven change from boreal to temperate ecosystems (Fig. 4). The creation of a mosaic of forests, pastures and arable fields in recent history has led to the unprecedented rise of palynologically inferred species richness in our record (Fig. 5). Tree species diversity, on the other hand, has steadily been decreasing since the Bronze Age, with the decline of many disturbance-sensitive taxa such as *Tilia*, *Ulmus* and *Acer*. However, the persistence of these diverse, mixed temperate-continental forests until well into the Late Holocene is remarkable. Central European sites situated on more fertile soils where Neolithic settlements are documented show a rapid decline of these disturbance-sensitive taxa already during the mid-Holocene, i.e. after ca. 6,500 cal bp (Rösch 1992; Kalis et al. 2003; Thöle et al. 2016; Rey et al. 2019), indicating that the impoverishment of the Central European temperate forest was primarily driven by anthropogenic disturbance.

## Conclusions

Our study shows that the area surrounding Lake Svityaz was always very marginal for land use compared to other regions of Europe, where intense agricultural activity was already causing dramatic forest vegetation shifts during the Neolithic (Fyfe et al. 2015; Roberts et al. 2018; Whitlock et al. 2018). While vegetation structure (e.g. forest density/openness) remained almost unaltered until ca. 500 years ago, forest composition changed 3,000–2,500 years ago, likely in response to intensified human impact. Recent anthropogenic disturbance during historic times (i.e. the last 500 years) led to an increase in overall plant diversity by creating new habitats, introducing new species, such as cultivated plants and weeds, and increasing ecosystem heterogeneity. For the greater part of the Holocene (ca. 10,000–3,000 cal bp), mixed temperate–continental forest dominated the area, with higher abundances than at present of light-loving temperate trees such as *Tilia*, *Ulmus*, *Acer* or *Corylus*. The longer persistence of such highly diverse mixed forest in an agriculturally marginal area indicates that human disturbance was a major driver of Central European forest dynamics in more fertile regions. The use of fire to clear forest for arable land, browsing by livestock and selective logging led to a decline of disturbance-sensitive taxa such as *Tilia*, *Ulmus* and *Acer.*

Our record clearly indicates that at Lake Svityaz, climate was the main driver of vegetation and fire dynamics until the Late Holocene. Slightly higher than present temperatures during the Holocene Thermal Maximum, possibly in combination with more flammable forests, led to a markedly higher fire activity in the region under near-natural conditions. This implies that warmer and drier conditions, as projected with future climate change, could significantly increase the risk of forest fires in the region. Indeed, the current climate warming has led to devastating fires in the temperate-continental forests of Ukraine, Belarus and Russia in recent years, which is of particular concern due to the presence of forests that are highly contaminated by radioactive fallout after the explosion of the Chernobyl nuclear power plant in 1986 (Evangeliou et al. 2015). The situation is further exacerbated by the extensive plantation of flammable pine in the region since the 1950s, which now cover more than 60% of the forested area in the Ukrainian Polesie alone (Moroz et al. 2020). A return to more mixed deciduous forests including *C. betulus*, *Tilia*, *Ulmus*, *Acer* and *Quercus* could reduce the fire risk due to lower flammability, which would be an important step toward climate change mitigation.

## Supplementary Information

Below is the link to the electronic supplementary material.

Supplementary file1 (TIF 1048 KB)

## Data Availability

All data is currently stored in the Alpine Pollen Database (ALPADABA) and will be made publicly available on the European Pollen Database (EPD, https://www.europeanpollendatabase.net) and Neotoma (https://www.neotomadb.org) upon publication of this article.
