# Supplementary figures and images for: Holocene vegetation, fire and land use dynamics at Lake Svityaz, an agriculturally marginal site in northwestern Ukraine
Source: Veg Hist Archaeobot. 2021 Jun 21;31(2):155–70. doi: 10.1007/s00334-021-00844-z (PMC8897337; doi:10.1007/s00334-021-00844-z)

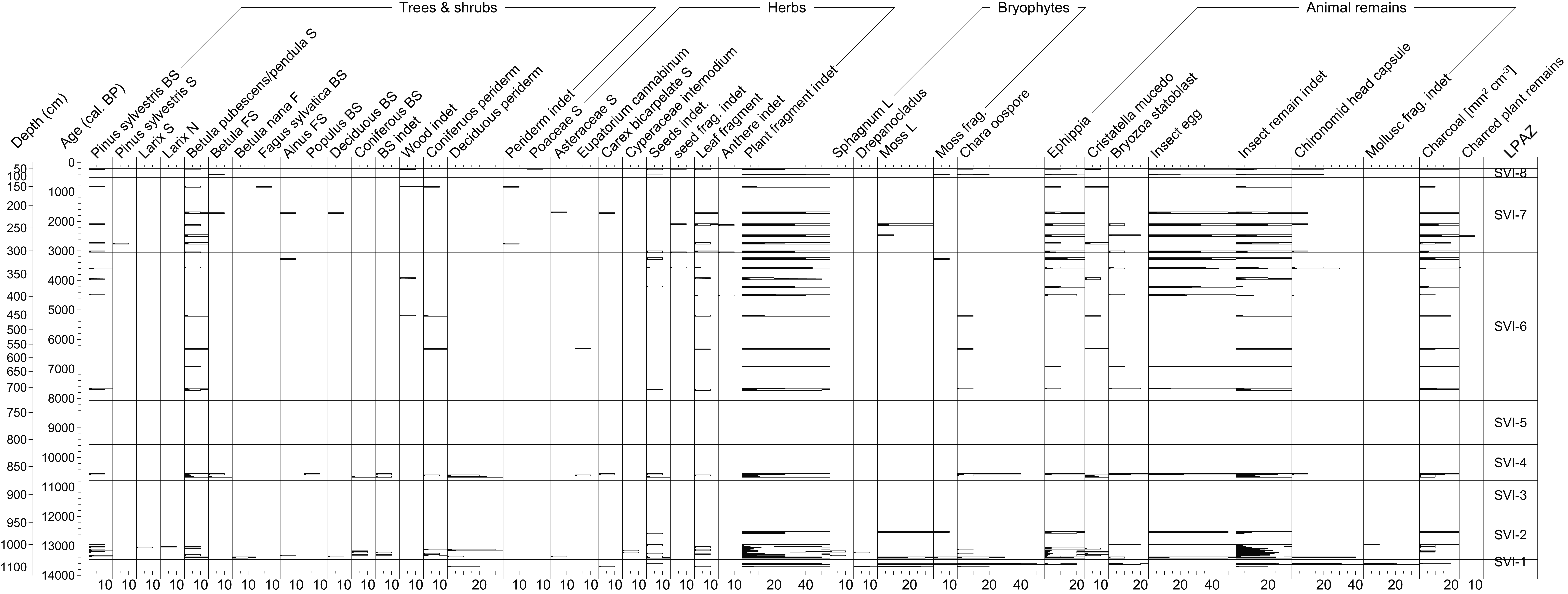

Supplement: Supplementary file 1 — Supplementary file1 (TIF 1048 KB) [file 334_2021_844_MOESM1_ESM.tif]
